# Stimulation with acoustic white noise enhances motor excitability and sensorimotor integration

**DOI:** 10.1038/s41598-022-17055-9

**Published:** 2022-07-30

**Authors:** Giovanni Pellegrino, Mattia Pinardi, Anna-Lisa Schuler, Eliane Kobayashi, Stefano Masiero, Gino Marioni, Vincenzo di Lazzaro, Flavio Keller, Giorgio Arcara, Francesco Piccione, Giovanni Di Pino

**Affiliations:** 1 Department of Neurology and Neurosurgery, Montreal Neurological Institute and Hospital, 3801 University Street, Montréal, QC H3A 2B4 Canada; 2 NeXT: Neurophysiology and Neuro-Engineering of Human-Technology Interaction Research Unit, Campus Bio-Medico University, Via Alvaro del Portillo, 200, 00128 Rome, Italy; 3 IRCCS San Camillo Hospital, Via Alberoni 70, Venezia Lido, Venezia, Italy; 4 Department of Neuroscience, University of Padua, Via Giustiniani, 5, 35128 Padova, Italy; 5 Section of Otolaryngology, Department of Neuroscience DNS, University of Padua, Via Giustiniani, 5, 35128 Padova, Italy; 6 Department of Neurology, Campus BioMedico University of Rome, Via Alvaro del Portillo, 200, 00128 Rome, Italy

**Keywords:** Sensorimotor processing, Motor cortex, Cortex, Excitability

## Abstract

Auditory white noise (WN) is widely used in neuroscience to mask unwanted environmental noise and cues, e.g. TMS clicks. However, to date there is no research on the influence of WN on corticospinal excitability and potentially associated sensorimotor integration itself. Here we tested the hypothesis, if WN induces M1 excitability changes and improves sensorimotor performance. M1 excitability (spTMS, SICI, ICF, I/O curve) and sensorimotor reaction-time performance were quantified before, during and after WN stimulation in a set of experiments performed in a cohort of 61 healthy subjects. WN enhanced M1 corticospinal excitability, not just during exposure, but also during silence periods intermingled with WN, and up to several minutes after the end of exposure. Two independent behavioural experiments highlighted that WN improved multimodal sensorimotor performance. The enduring excitability modulation combined with the effects on behaviour suggest that WN might induce neural plasticity. WN is thus a relevant modulator of corticospinal function; its neurobiological effects should not be neglected and could in fact be exploited in research applications.

## Introduction

Acoustic white noise (WN) is widely applied in neuroscience experiments to mask unwanted sounds. In the field of neurophysiology, for example, WN is applied to mask the stimulation coil click during simultaneous transcranial magnetic stimulation/electroencephalography (EEG-TMS) studies^1^. TMS-evoked potentials, which are the potentials evoked by TMS and recorded by EEG during EEG-TMS, allow to characterize cortical function^2,3^, but are hampered by several confounding factors, including the TMS clicking sound^1^. The latter scales with the intensity of the stimulator output and produces auditory evoked potentials with a latency of about 100 and 180 ms, potentially aliasing the effects of the stimulation^4,5^. Click-related auditory potentials are typically mitigated by delivering WN auditory stimulation during TMS-EEG^6–16^. More generally, WN is also exploited to mask unwanted auditory cues and environmental noise, or as a control condition for auditory tasks in experimental neuroscience^17–23^. These masking approaches, however, neglect the potential influence of WN on cortical and corticospinal excitability as well as behaviour.

Previous evidence suggests that cortical function is continuously modulated by a stream of endogenous and external signals: watching a silent movie reshapes brain networks^24^, smelling food increases motor cortex excitability^25^, and spontaneous fluctuations of vigilance strongly condition cortical connectivity^26,27^.

It is therefore not surprising that WN impacts on cortical function and has been exploited for a variety of interventional and therapeutic applications. As early as 1667, sculptor and architect Gian Lorenzo Bernini invented a WN sleeping machine for treating Pope Clement IX’s insomnia (Giulio Rospigliosi, 1600–1669)^28^. Today WN stimulation is delivered to promote sleep^29–32^, improve learning and perception^33–36^, treat tinnitus^37,38^, as well as postural control^39–41^.

The action mechanisms of WN are however poorly understood. It is possible that some of the effects of WN could be interpreted in the framework of the stochastic resonance theory, which posits that noise not carrying specific information has the potential to modulate brain activity^42^. Based on this theory, the introduction of WN into a non-linear system (e.g. brain) leads to synchronization of neurons and, in turn, to improved processing of sensory inputs^43^. In this respect transcranial random noise stimulation (tRNS) has also been shown to increase corticospinal excitability^44^.

Based on these preliminary observations of WN exposure having potential widespread cortical effects, we expected WN exposure to increase M1 corticospinal excitability and improve sensorimotor performance. Hence, we measured motor evoked potentials (MEPs) from single pulse TMS (spTMS) before, during and after WN exposure to verify its effect on corticospinal excitability; moreover, we evaluated the TMS I/O curve (100, 120 and 140% of resting motor threshold, rMT) and short-latency intra cortical inhibition (SICI) and intra cortical facilitation (ICF) paradigms. Finally, to test whether WN generally influences the sensorimotor circuit or has a specific effect on M1, we assessed sensorimotor (reaction time tasks) and pure motor (dexterity and force tasks) facilitation.

## Materials and methods

### Participants and experimental design

This study was approved by the Research Ethics Committees of Province of Venice and of the Università Campus Bio-Medico di Roma. Experimental procedures followed the 1964 Helsinki declaration and its later amendments. Subjects signed a written informed consent prior to participation.

The study included 61 healthy subjects taking part in one of three experiments (i.e., excitability experiment, behavioural experiment, and web-based behavioural experiment). All participants were aged 20–50, had normal hearing, no present or past history of neurological or psychiatric disorders, and none of them was taking central nervous system active medications.

### Excitability experiment

**Figure 1:**
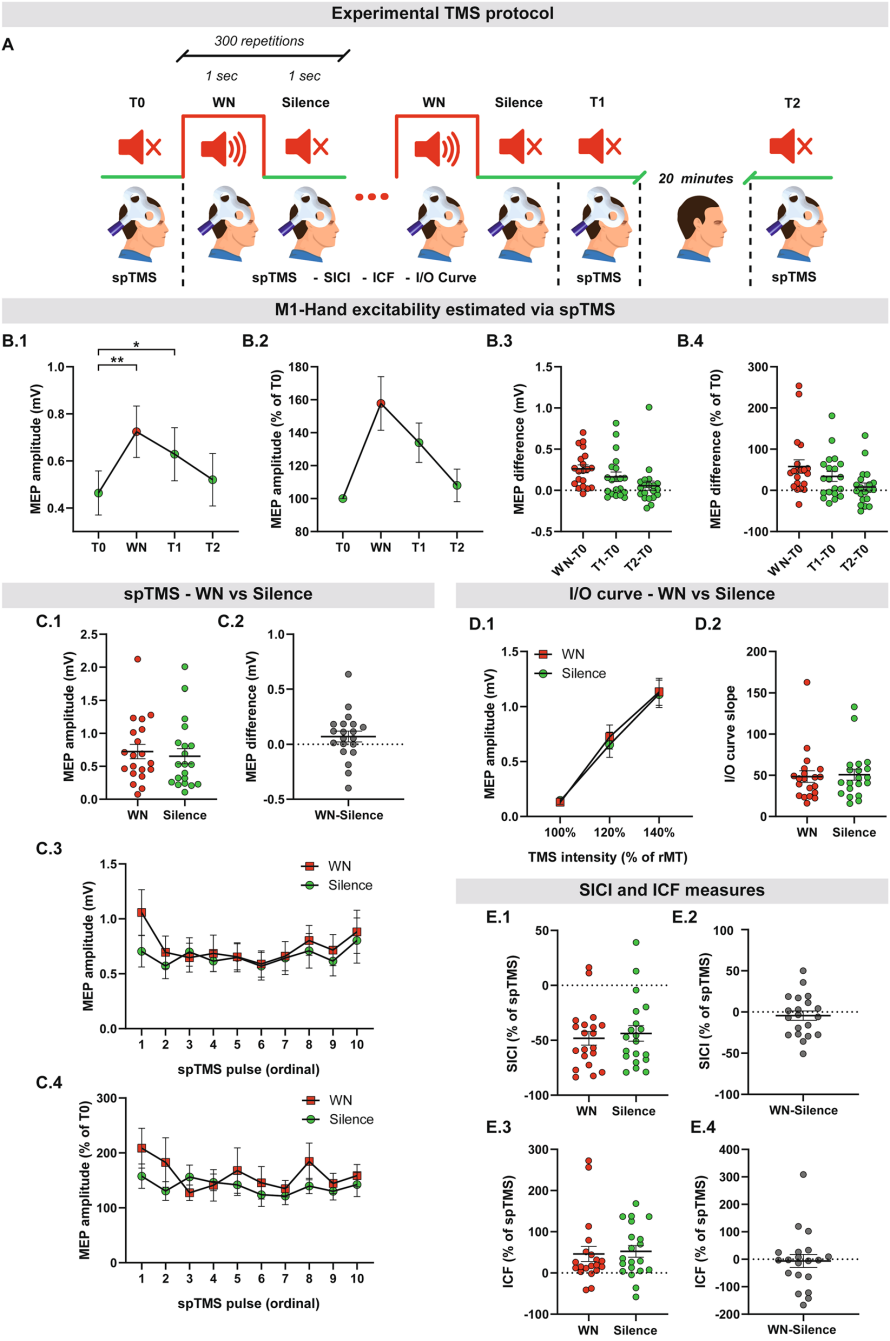
Cortical and Corticospinal Excitability. (**A**) Experimental design. Subjects were exposed to a sequence of WN (1 s) interleaved with Silence (1 s), repeated 300 times. Corticospinal excitability was assessed for the hotspot of the right M1-Hand abductor digiti minimi (ADM) at multiple time-points: before WN exposure (T0), during WN (WN), during silence (Silence), immediately after the end of the sequence (T1) and 20 min later (T2). Cortical excitability was assessed during WN (WN) and during silence (Silence). spTMS = single pulse TMS, rMT = resting motor threshold, SICI = short-latency intra cortical inhibition, ICF = intra cortical facilitation, I/O curve = input/output curve. (**B**) M1-Hand excitability estimated via spTMS. As compared to T0, corticospinal excitability was significantly higher during WN exposure (WN about 60% higher than T0) and soon after the WN-Silence sequence (T1, about 40% higher than T0). Statistical analysis was performed only on data illustrated in (**B1**), while (**B2**), (**B3**) and (**B4**) are shown for visualization purpose only. (**B1**) absolute MEP amplitude; (**B2**) MEP amplitude as percentage of T0. (**B3**) Individual absolute MEP difference between conditions. (**B4**) Individual normalized MEP differences between conditions. (**C**) There was no significant spTMS excitability difference between WN and Silence. (**C1**) Individual absolute MEP amplitude for WN and Silence. (**C2**) Individual WN-Silence absolute MEP difference. (**C3**) Absolute MEP amplitude over the duration of WN and Silence. The x-axis indicates the sequence of TMS pulse. Corticospinal excitability level remained stable during the entire auditory exposure and did not show any online significant difference between WN and Silence. Data is reported as average ± SEM across subjects. (**C4**) As for (**C3**), but data was normalized over T0 excitability. (**D**) There was no significant I/O curve difference between WN and Silence (**D1**) Absolute MEP amplitude at different TMS intensities for WN and Silence. (**D2**) Individual values of I/O curve slope for WN and Silence. (**E**) SICI and ICF were not significantly different between WN and Silence. (**E1**) SICI individual values for WN and Silence. (**E2**) SICI WN-Silence intrasubject difference. (**E3**) ICF individual values for WN and Silence. (**E4**) ICF WN-Silence intrasubject difference. Variability is expressed as standard error of the mean. * denotes p < 0.05; ** denotes p < 0.001.

Twenty subjects (mean age = 28.55 ± 5.52 (St.Dev.); 8 F; Oldfield’s Edinburgh Inventory: 69.94 ± 21.54)^45^ were recruited for the M1 excitability experiment, which was the first to be performed (Fig. 1A). Positive Edinburgh scores express participant’s right handedness, while negative scores express left handedness. Complete preference for right hand or left hand are expressed by a score of 100 or − 100, respectively.

Participants were comfortably seated on an armchair with padded armrests and were instructed to relax for the entire duration of the experiment. WN was delivered binaurally via earplugs, at 85 dB sound pressure level^46–50^. We opted for an interrupted time-series experimental design. Participants were exposed to an auditory sequence consisting of 300 blocks of 1 s WN (*WN*) interleaved with 1 s silence (Silence). Corticospinal excitability (MEPs) was measured during both WN and Silence blocks, at several time points in relation to WN exposure: prior (*T0*), during (*WN*), immediately after (*T1*) and 20 min following auditory WN exposure (*T2*).

Corticospinal excitability was evaluated with a BiStim^2^ TMS stimulator (The Magstim Co. Ltd) connected to a 70 mm eight-shaped coil, set to deliver a monophasic pulse and to induce a current flowing in the posterior-to-anterior direction across the central sulcus. We targeted the right M1 cortex because it was shown that the dominant hemisphere in right-handed individuals (i.e. left cortex) has higher excitability compared to the non-dominant one (i.e. right cortex). As we aimed to test the effect of WN on corticospinal excitability, targeting the non-dominant hemisphere prevented possible bias^51^. The stimulation targeted the hotspot for the *abductor digiti minimi* (ADM) muscle of the left hand, located in the right M1 hand region. We opted for investigating ADM muscle as a previous study^52^ demonstrated that cortical representation of ADM is slightly more medial than FDI (higher distance from the primary auditory cortex). As such, ADM may provide a more specific readout of A1-M1 interactions, being less influenced by the spread of A1 activation to neighbour areas. The TMS coil was handheld with a direction perpendicular to the direction of the precentral gyrus^52^. EMG was recorded from the left ADM muscles with disposable surface paramagnetic circular electrodes and applying a standard belly-tendon montage. EMG signal was amplified, bandpass filtered (20 Hz–3 kHz) and digitized at 5 kHz with a CED 1401 system equipped with the Signal software (Cambridge Electronic Design, Cambridge, UK). The hotspot was defined as the optimal scalp position for eliciting MEPs of maximal amplitude in the contralateral ADM^53^. rMT was estimated at T0 with the Maximum-Likelihood strategy^54^. TMS was never delivered within 300 ms from the transition between WN and Silence to avoid potential multisensory interactions between auditory transition and stimulation. Multisensory interactions between stimuli are indeed bound to occur in a tight time-window of 120 ms from stimulus onset^55^. Finally, TMS pulses were delivered with a jittered interval ranging between 5000 and 7000 ms.

The following measures of corticospinal and cortical excitability were collected:

- *MEP amplitude after spTMS*, which provides a global measure of corticospinal excitability. The stimulation intensity was set to 120% of the T0-rMT. Twenty spTMS pulses were given at time-points T0, T1 and T2. No WN stimulation was delivered during these time-points.

In order to assess the *online* effects of WN on *specific* intracortical excitatory and inhibitory circuits we further evaluated:

(b) *Input/output recruitment curve (I/O curve), which* investigates changes of MEP amplitude as a function of the stimulation intensity. The slope of the I/O curve is an indirect measure of glutamatergic transmission^56,57^. Three stimulation intensities were considered for this purpose: 100% T0-rMT, 120% T0-rMT and 140% T0-rMT^58^. Ten TMS pulses were given for each intensity during both Silence and WN^58,59^.
(c) *SICI and ICF*, which provide an indirect estimation of intracortical inhibitory circuits (GABAergic transmission) and excitatory circuits, respectively^60^. SICI and ICF are assessed with paired pulse TMS (ppTMS). The first pulse is a conditioning stimulus and the second one is the test stimulus^61^. For the SICI protocol, the conditioning stimulus intensity was 90% of T0-rMT^62^, the test stimulus intensity was 120% of T0-rMT and the inter-stimulus interval (ISI) was 2 ms. For the ICF protocol, the conditioning stimulus intensity was 90% of T0-rMT^63^, the test stimulus intensity was 120% of T0-rMT and the ISI was 11 ms. SICI and ICF were expressed as percentage of spTMS excitability^64^, i.e. the difference between the two respective values was divided by the mean of the two values and multiplied by 100. SICI and ICF were measured with 20 ppTMS during Silence and 20 ppTMS during WN.

### Behavioural experiment

The behavioural study included 20 participants (10 females, mean age 25.70 ± 4.57, Oldfield’s Edinburgh Inventory: 77.95 ± 21.08). The behavioural experiments were designed based on the results of our TMS experiment, which showed a modulation of corticospinal excitability during and following WN exposure.

We concentrated on two tasks that investigate the influence of WN on motor abilities and sensorimotor integration:

### Hybrid reaction time task and maximum finger force and finger abduction dexterity task

**Figure 2:**
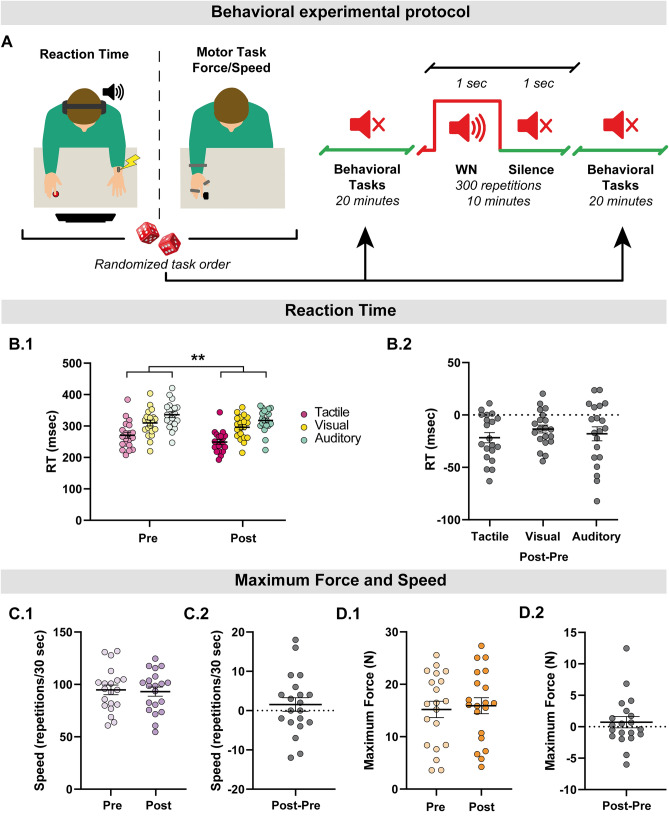
Effects of WN on behaviour. (**A**) Schematic representations of the behavioural tasks. The order of the tasks was randomized across subjects but kept constant within subject pre and post WN. The Maximum Finger Force Task and Finger Abduction Dexterity tasks aimed at assessing WN effects on pure, self-paced and self-initiated motor performance. The Maximum Finger Force Task consisted in pressing with maximal strength the right index finger against a force sensor. The Finger Abduction Dexterity Task consisted in abducting as fast as possible the right index finger. The Hybrid Reaction Time Task was meant to assess WN effects on complex sensory-motor integration. Subjects were cued through three different sensory routes (visual, auditory, tactile) to press as quickly as possible a keyboard key with their right index finger. They were seated on an armchair, had in-ear headphones, and median nerve skin electrodes connected to a stimulator. Subjects were asked to fixate a cross. The visual cue was a red circle, which disappeared at the time of the keyboard press; the acoustic cue was a pure sound; the tactile stimulus was an electric pulse. Thirty stimuli for each stimulus type were delivered in a random order. (**B**) WN significantly improved the Hybrid Reaction Time Task performance, similarly for the three tasks. Statistical analysis was performed only on data illustrated in (**B1**), while (**B2**) is shown for visualization purpose only. (**B1**) Individual performance for tactile, visual and auditory RT tasks. (**B2**) Intrasubject Post vs Pre difference in performance for tactile, visual and auditory RT tasks. (**C**,**D**) WN had no significant effects on Maximum Finger Force Task and Finger Abduction Dexterity Task performance. All panels show the individual performance for WN and Silence and the intrasubject WN-Silence performance difference. (**C1**) Speed individual values. (**C2**) Speed Post–Pre intrasubject difference. Variability is expressed as standard error of the mean. (**D1**) Maximum Force individual values. (**D2**) Maximum Force Post–Pre intrasubject difference. ** denotes p < 0.001.

These two tasks were performed before and after exposure to WN. WN was delivered as in the Excitability Experiment, i.e. the auditory sequence consisted of 300 blocks of 1 s of WN interleaved with 1 s of Silence:

- *Hybrid reaction time task.* This task was meant to assess WN effects on simple sensorimotor integration. Subjects were cued through three different sensory routes (visual, auditory, tactile) to press as quickly as possible a keyboard key with their right index finger. They were seated on an armchair, at approximately 65 cm from a 24" screen (DELL, 1600 × 900p resolution), had in-ear headphones (Sony MDR-EX15LP), and two skin electrodes at the wrist in the vicinity of the median nerve connected to a stimulator (Digitimer Electrical Stimulator—DS7A). Subjects were asked to fixate a black cross displayed on a white background. The visual cue was a red circle, which disappeared after keypress; the acoustic cue was a pure sound at 1000 Hz frequency and 50 ms duration; the tactile stimulus was a square electric pulse with an intensity of twice the subject’s sensitive threshold and 200 μsec duration. Thirty stimuli were delivered for each stimulus type, in a random order. The ISI ranged randomly between 1.5 and 2.5 s^65^ (Fig. 2A). Since the task involved three different sensory routes (visual, acoustic and tactile) and participants had to respond to each stimulus with a button press, we considered three different reaction time measures: visuo-motor, auditory-motor and tactile-motor.
- *Maximum finger force task and finger abduction dexterity task.* These tasks aimed at assessing WN effects on pure, self-paced and self-initiated motor performance. The *Maximum Finger Force Task* consisted in pressing with maximal strength the right index finger against a force sensor. The outcome measure was the average force over three repetitions. The *Finger Abduction Dexterity Task* consisted in abducting as fast as possible the right index finger. The outcome measure (Speed) was the number of repetitions in 30 s. For both tasks, subjects were comfortably seated at a table, and had their right forearm held with a velcro strap on a custom-made wooden support (Fig. 2A).

The order of the tasks was randomized across subjects and kept constant within subject pre and post WN.

### Web-based behavioural experiment

**Figure 3:**
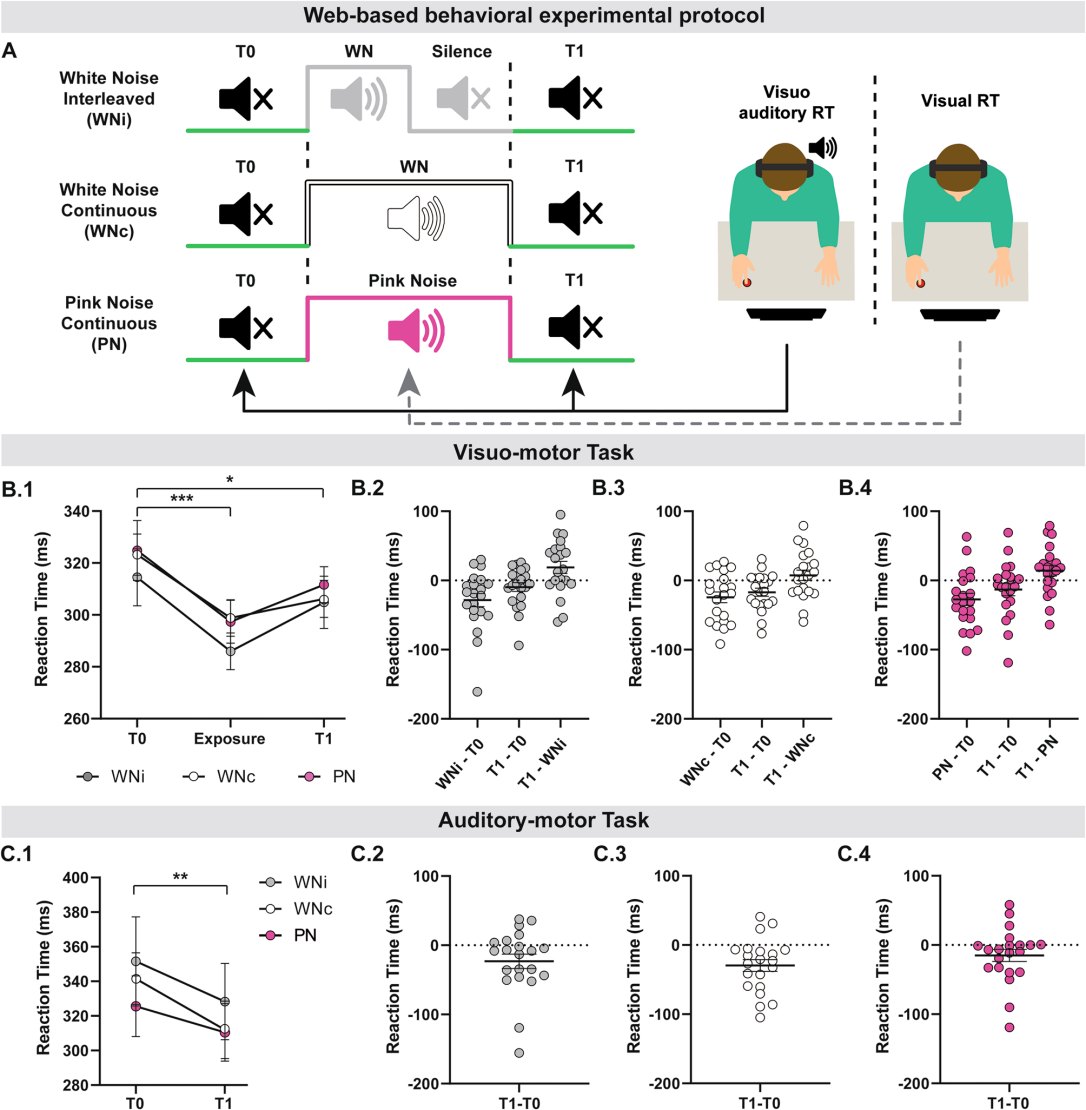
Web-based behavioural experiment. (**A**) Schematic representation of web-based behavioural task. This experiment was designed to assess WNi, (White Noise Interleaved), WNc (White Noise Continuous) and PN (Pink Noise) effects on complex sensory-motor integration. The order of conditions was randomized across subjects. Subjects were cued through two different sensory routes (visual, auditory) to press as quickly as possible a keyboard key with their right index finger. Visuo-motor RT was performed at T0, during auditory exposure and at T1. Auditory-motor RT was performed at T0 and T1. Subjects were at home, seated in front of their PC screen with earplugs/phones. Subjects were asked to fixate at a cross. The visual cue was a red circle; the acoustic cue was a pure sound. (**B**) Visuo-motor task. WNi, WNc and PN improved performance of visuo-motor task, both during and after exposure to auditory noise. (**B1**) Mean ± SER performance across subjects, by condition (WNi, WNc, PN), before (T0), during (Exposure) and after (T1) exposure to auditory noise. (**B2**) WNi intrasubject RT differences. (**B3**) WNc intrasubject RT differences. (**B4**) PN intrasubject RT differences. (**C**) Auditory-Motor task. WNi, WNc and PN improved performance of auditory-motor task. (**C1**) Mean ± SER performance across subjects by condition (WNi, WNc, PN), before (T0) and after (T1) exposure to auditory noise; (**C2**) WNi intrasubject RT difference; (**C3**) WNc intrasubject RT difference; (**C4**) PN intrasubject RT differences. Variability is expressed as standard error of the mean. *,** and *** denote p < 0.05, p < 0.001 and p < 0.001 respectively.

An additional web-based behavioural experiment was conducted with two objectives: (i) to test whether continuous and interleaved WN exposures affect brain function differently; (ii) to test whether WN and pink noise have different effects on brain function. This experiment included 21 participants (12 females, mean age 29.32 ± 6.53, Oldfield’s Edinburgh Inventory: 71.25 ± 20.77). Contrarily to WN, which has equal intensity at all frequencies, pink noise follows the 1/f law, with a power spectral density inversely proportional to frequency (higher power for lower frequencies and lower power for higher frequencies). Acoustic pink noise has a very simple structure and no rich frequency modulation. Subjects had to complete a visuomotor *Reaction Time Task* (RT) before, during and after auditory exposure. We opted for a randomized, paired design, with each participant tested under three conditions: (i) *continuous WN*; (ii) *interleaved WN* (1 s of WN and 1 s of Silence); (iii) *continuous Pink Noise*. Sessions were performed in three different days, approximately at the same time of the day. Each auditory exposure lasted 10 min, consistent with the duration of auditory exposure of the TMS and in lab behavioural experiments. This experiment was coded with PsychoPy v3.0^66,67^ and ran through Pavlovia platform (https://pavlovia.org/). The source code is freely available at https://github.com/giorgioarcara/MEG-Lab-SC-code/tree/master/WHITE-NOISE/WN_Psychopy_Task_ver3. Subjects were instructed to comfortably seat in front of their computer monitor, at home, in a quiet environment. They were asked to wear headphones/earplugs and to remain focused for the entire duration of the experiment. The details of the experimental design are available in Fig. 3A. Briefly, auditory intensity at earphones/earplugs was fine-tuned for each subject with an online hearing threshold staircase (see Supplementary Material). The intensity was ultimately set to the mid value between maximum volume and auditory threshold and roughly corresponded to 65 dB. Subjects were asked to fixate a black cross displayed on a white background for the entire experiment and to perform an auditory/visuo-motor reaction time task before and after auditory exposure, and a visuo-motor reaction task during auditory exposure (visual route only, as they were being exposed to auditory noise). The latter task started 2 min after auditory exposure and lasted 1 min. Thirty stimuli were given for each sensory route, in a random order, with an ISI ranging between 1.6 and 2.4 s. Visual stimulus never appeared within 300 ms from the transition between WN and Silence of the interleaved exposure. The visual cue was a red circle, the acoustic cue was a pure sound at 1000 Hz lasting 50 ms. Participants had to press a keyboard key as quickly as possible with their right index finger following the cue. The outcome measure was the time elapsed between cue and button press. Further details are available in the Supplementary Material.

### Statistical analyses

Statistical analysis was performed with IBM SPSS (Ver. 24).

- To test whether there was any WN-related modulation of global corticospinal excitability, we evaluated spTMS measures (peak-to-peak amplitude) and computed a repeated measures ANOVA with 4 levels (T0, WN, T1, T2). We compared WN, T1 and T2 *versus* T0, by running post-hoc t-tests, which were Bonferroni corrected.
- To test whether there were excitability variations, which were transient and temporally restricted to the time of WN exposure, spTMS, I/O recruitment curves and SICI and ICF were directly contrasted between WN and Silence with a two-sided paired sample t-test. For I/O recruitment curves comparison we calculated the slopes of the respective curves, since they could be approximated to a straight line, and fed the slope as dependent variable into the statistical model. The significance level was set to p < 0.05 and Bonferroni correction was applied.
- For *Hybrid Reaction Time Task* and *Web-based behavioural experiment* we performed repeated measures ANOVA. Post-hoc analyses were performed through Bonferroni corrected paired t-tests. For *Maximum Finger Force Task and Finger Abduction Dexterity Task* paired t-tests were applied.

### Ethics approval

This study was approved by the Research Ethics Committees of Province of Venice and of the Università Campus Bio-Medico di Roma. Experimental procedures followed the 1964 Helsinki declaration and its later amendments.

## Results

### Excitability experiment

WN induced a significant increase of spTMS corticospinal excitability, lasting up to several minutes after the end of auditory exposure (T1). At T2, mean corticospinal excitability was still higher than T0, but this difference was not statistically significant (*Repeated measures ANOVA F(3,57)* = *11.489, p* < *0.001; post-hoc T0 vs WN p* < *0.001, T0 vs T1 p* = *0.018, T0 vs T2 p* > *0.200*) (Fig. 1B).

Silence and WN did not show any significant difference in corticospinal excitability, as evaluated by spTMS, SICI and ICF, as well as the I/O recruitment curves (p > 0.100 consistently) (Fig. 1C–E). In order to investigate, if there was an effect of habituation or sensitization due to stimulation, we investigated the mean amplitude of MEP for WN and Silence (Fig. 1C.3,C.4). There was no significant increase or decrease of MEPs as a factor of time.

### Behavioural experiments

The performance improved significantly after WN for the *Hybrid Reaction Time Task (repeated measures ANOVA, main factor Time: F(2, 38)* = *82.614, p* < *0.001)* (Fig. 2B)*.* The effect was similar across tasks, as there was no significant *Time by Task* interaction *(p* > *0.200). Maximum Finger Force Task* and *Finger Abduction Dexterity Task* did not significantly change after WN as compared to before (two-tailed paired sample t-tests p > 0.100 consistently, Fig. 2C,D.

### Web-based behavioural experiment

Auditory exposure significantly modulated visuo-motor RT, regardless of the type of auditory noise (*repeated measures ANOVA with factors Condition: Pink Noise, WN interleaved, WN continuous; Time:T0, Exposure, T1; Factor Time F(2, 40)* = *9.702, p* < *0.001; no significant main factor Condition or Condition by Time interaction*). Post-hoc analysis demonstrated that, as compared to *T0*, RT was lower during (*Exposure*) and after (*T1*) auditory exposure (*p* < *0.001 and p* = *0.025, respectively*). Figure 3B. A similar behaviour was observed for the auditory-motor RT task, with RT being significantly lower at *T1* as compared to *T0* (*Repeated measures ANOVA with factors Condition: Pink Noise, WN interleaved, WN continuous; Time:T0, T1; Factor Time F(1, 20)* = *11.094, p* = *0.003; no significant main factor Condition or Condition by Time interaction;* Fig. 3C).

## Discussion

Despite its broad application in neuroscience and neurostimulation experiments, the influence of acoustic WN per se on corticospinal excitability has never been taken into account. Here we show that M1 corticospinal excitability increases during and after WN exposure. It has been shown before that M1 corticospinal excitability increases while listening to meaningful sounds and speech^68,69^. For instance, listening to music modulates the excitability of M1 areas mapping for specific groups of muscles^70^; listening to speech increases the excitability of the left (language dominant) M1, with greater impact on regions subserving muscles recruited during speech^71^. A tight connection between A1 and M1 has been shown in previous studies^21,72–74^. Moreover, an fMRI study has unveiled a WN-related connectivity increase between subcortical dopaminergic nuclei and right superior temporal sulcus^34^, potentially hinting towards the influence of WN on motor planning. A general connection between motor and auditory networks could be shown during continuous theta burst stimulation (cTBS)^65^ over the right auditory cortex^75^. The increase in MEPs during and after WN exposure in our study further underlines this strong connection and provides for the first time direct evidence of increased M1 excitability due to WN exposure.

Importantly, the repetition of WN sequences (1 s) resulted in *remarkable (spTMS 40–60%)* excitability increase in M1 as compared to baseline acquisition, outlasting the acute noise exposure. This effect turned out to be stable over the sequence of single pulses and is therefore not an effect of sensitization (compare^76^). Beyond that, global excitability (spTMS) increase remained higher than baseline until 20 min after the end of auditory exposure (T2), with a significant effect for several minutes after stimulus presentation (T1) (Fig. 1B).

The interleaved WN and Silence acts as a sequence or pattern during which the excitability (spTMS, SICI, ICF, I/O curve) remains stable, as we did not find any difference between these two conditions. The study design did not include an assessment of SICI and ICF before and after exposure to the noise (Fig. 1), therefore we are not able to make any claim on the influence of WN on intracortical circuitry (SICI, ICF, I/O curve) beyond the comparison between WN and Silence during the stimulation phase. This will be addressed in future studies. Taken together, the persistence in excitability, in combination with the behavioural results (see below), suggest activation of Long-Term Plasticity-like (LTP-like) mechanisms due to WN exposure^53,60,77^. While we focused on excitability estimated from ADM MEPs, future studies should more accurately map the effects of WN on the excitability profile of the entire primary motor and premotor cortex^52^.

Listening to WN might promote on-line behavioural improvement through a mechanism known as stochastic resonance or stochastic facilitation^78^ (for a review the reader is referred to^42^). Stochastic resonance postulates that the random probability distribution of noise structure can enhance neural processing for some tasks, dependent on attention level, noise intensity etc.^34,78–84^. Previous examples of behavioural improvements driven by ‘noise’ within the stochastic resonance framework exist. In particular, transcranial random noise stimulation (tRNS) is a non-invasive brain stimulation technique employing random noise electrical currents applied to cortical surface through the scalp. The modulation of cortical excitability and plasticity^85,86^ and the behavioural improvement^87–89^ induced by tRNS in healthy subjects and patients^90^ are paradigmatic examples of functional changes due to noise stimulation and related to stochastic resonance.

No significant off-line effects were found for pure motor tasks. Conversely, a statistically significant improvement occurred in all tasks for the sensorimotor domain. The improvement in performance was independent of the sensory route^91,92^. This demonstrates that changes in excitability can result in improved behaviour occurring minutes after WN exposure, in agreement with the idea that WN induces plasticity effects. Moreover, the absence of effects on pure motor tasks, suggests that sensorimotor integration may be the domain more sensible to the induction of WN-associated plasticity, underlining that WN effects reverberate up to multiple and distant brain networks.

The web-based behavioural experiment further underlined that WN improves performance in visuo-motor and auditory-motor reaction time. More importantly, this experiment confirmed that continuous and interleaved WN (here denoted as WNc and WNi) have similar behavioural effects, which outlast the duration of auditory noise stimulation. The improvement of performance also occurred during auditory exposure (online), consistently with our findings from the excitability experiment, and potentially supported by stochastic resonance. Finally, no significant differences were found between WN and pink noise (more often encountered in real life), suggesting that auditory noises with similar simple structure may share similar effects. Note that no correlation analysis could be performed between neurophysiological measures and behavioural measures as we recruited two independent groups, but future studies would benefit from an intra-subject design and assess the excitability-behavioural relationship. Neurophysiological measures refer to the right non-dominant hemisphere, whereas behavioural measures are mostly linked (for the motor output) to the left dominant hemisphere. We do not expect this to be a remarkable limitation for the interpretation of our results, especially considering that WN is represented bilaterally^49^.

Concerning the duration of the effects induced by exposure to noise, we did not find a significant excitability increase at T2, therefore the aftereffects on corticospinal excitability observed here lasted only for a few minutes after the end of exposure to WN. There has been a long debate on the duration of the excitability after-effects and how much and under what circumstances they reflect plasticity phenomena. It has been learned that they can be short lasting, depending on multiple factors. For example, the seminal work on tDCS performed with 1 mV intensity and 5 min duration induced an excitability increase lasting about 5 min and getting back to baseline within roughly 10 min^93^. Changing stimulation parameters may prolong the effects for up to 1.5 h^94,95^. A similar pattern has been observed for other well-established plasticity-inducing non-invasive brain stimulation approaches (for a review the reader is referred to^96^). tRNS has a rather immediate effect on excitability, which soon reaches the steady state and lasts for up to one hour^85,97^. Similarly to tDCS, the dose of stimulation has an impact on the duration of tRNS after-effects^85^.

In our work, the increase of cortical excitability did not last long, but the behavioural experiment revealed online and offline effects up to 20 min after the end of the auditory noise. We did not test different parameters, duration, patterns of auditory exposure and the multiple sources of variability on cortical plasticity^98,99^: this will be the focus of our future work on the topic.

Over the past years, there has been a great interest in the link between auditory cortices and motor cortices. The interaction between auditory and motor cortices has been associated with functions such as speech, music processing and working memory^100–105^. Our focus here has been on this interaction in the attempt to understand if, how and under what circumstances the interplay between A1 and M1 could be exploited for modulating brain function, and obtaining lasting effects. Previously, we focused on the effects of modulation of M1 excitability upon auditory excitability and processing (Motor to Auditory) demonstrating that the effects of M1 inhibition reverberate up to the temporal cortex and can be unveiled with magnetoencephalography auditory steady state responses^50^. The auditory to motor interaction could be more translationally relevant, as one could imagine exposing subjects to auditory sounds/noises to achieve a useful modulation of motor cortex function. As outlined in the introduction, this is already performed in a range of fields, with for instance improvement of postural control^106,107^. The mechanisms by which auditory to motor interactions take place are however poorly understood. We have recently investigate d the online effects of exposure to continuous WN and richer noises such as the noise produced by an MR scanner on activity and connectivity of resting state networks and demonstrated that WN reduces connectivity of the motor network with other cortical regions^49^. We have also demonstrated that the online effect of these sounds is not limited to cortical activity, but most likely involves subcortical regions and results in an online change of autonomic function, as assessed by heart rate variability^48^. In the same line, we have started investigating the remote effects of auditory stimulation with 40 Hz amplitude modulated tones and demonstrated that there is a definite interaction between the auditory cortex and the premotor cortices and that such an interaction depends on cortical structure, more specifically on cortical thickness^108^. In our opinion, however, the most interesting auditory exposure patterns are those which have similarities with non-invasive brain stimulation applications, as these may induce lasting modulation of cortical function. In this perspective, this study provides the first and provisional evidence that auditory WN may share some similarities with random noise electrical stimulation. Further studies are needed to compel our results and to clarify mechanisms and parameters of stimulation, which are currently unknown. Should however these findings be confirmed, they could pave the way to auditory exposure as a potential neuromodulation intervention.

## Conclusions

Stimulation with acoustic WN has an enduring effect of increasing motor cortex excitability and enhances performance in sensorimotor integration. We suggest taking the potential effects of WN on M1 excitability into account while conducting experiments that use WN to mask other unwanted sounds, e.g. during TMS/EEG experiments.

## Supplementary Information

Supplementary Information.

## Data Availability

Data and code are available upon reasonable request addressed to the corresponding authors. Code for the web-based experiment is freely available under: https://github.com/giorgioarcara/MEG-Lab-SC-code/tree/master/WHITE-NOISE/WN_Psychopy_Task_ver3.
