# Supplementary material for: Stimulation with acoustic white noise enhances motor excitability and sensorimotor integration
Source: Sci Rep. 2022 Jul 30;12:13108. doi: 10.1038/s41598-022-17055-9 (PMC9338990; doi:10.1038/s41598-022-17055-9)
Supplement: Supplementary file 1 — Supplementary Information. [file 41598_2022_17055_MOESM1_ESM.pdf]

Title: **Stimulation with acoustic white noise enhances motor excitability and sensory-motor integration**

Authors: Giovanni Pellegrino*, MD, PhD[1], Mattia Pinardi*, PhD[2], Anna-Lisa Schuler, PhD[3], Eliane Kobayashi, MD, PhD[1], Stefano Masiero, MD[4], Gino Marioni, MD[5], Vincenzo di Lazzaro, MD[6], Flavio Keller, MD, PhD[2], Giorgio Arcara, PhD[3], Francesco Piccione, MD[3], Giovanni Di Pino, MD, PhD[2]

Authors marked with * equally contributed to this work.

Supplementary data

Web-Based experiment – instructions displayed on video

Web-based experiment. During the online behavioural experiment, subjects were guided by step-by-step on-screen instructions. The code of the experiment is freely available here: https://github.com/giorgioarcara/MEG-Lab-SC-code/tree/master/WHITE-NOISE/WN_Psychopy_Task_ver3.

Procedure to set auditory intensity at home

As the experiment was performed at home with different equipment by each participant, we had to adopt a protocol to standardize the sound intensity across subjects. The experiment therefore included a staircase procedure to set the sound intensity on an individual basis. Participants started by setting the PC/laptop volume to the maximum. Psychopy routine delivered a pure tone at 1000 Hz, lasting 50 ms, at 50% intensity. Participants were asked to recursively press down-arrow or up-arrow key depending on whether they heard or not the sounds. Sound intensity was automatically adjusted depending on subject's feedback. The steps for intensity adjustment were set on a linear scale. Auditory threshold was computed after 100 trials and the intensity for the experiment was set as the mid value between minimum hearable volume and initial (maximum) volume. On a pilot sample of ten subjects testing the procedure with different equipment (laptop, earplugs/phones, earplugs), the average intensity was about 65 dB.

Instructions displayed on video

During the staircase procedure, subjects were presented with the following text:

"*Increase the sound volume of your headphones to the maximum. Press DOWN arrow if you heard the sound or press UP arrow if you did not hear the sound.*"

At the beginning of the experiment, subjects were presented with the following text:

"*You will see a black cross in the centre of the screen. Keep your sight focused on the cross. A red dot will randomly pop up where the cross is, or a brief sound will be played. In both cases you will have to respond by pressing the Spacebar as fast as you can when one of the two stimuli (dot or sound) appears. After this task you will be prompted with new instructions. Press Spacebar to continue.*"

After this message, the first step of the experiment begun (T0), and subjects performed the Audio-Visual Reaction Time Task.

Then the following was presented:

"Well done! Now you will see the black cross once again. Keep your sight on the cross and relax. An audio track will be played in the background, but you are not required to do anything. At a certain point, the red dot will randomly appear again. When it will appear, press the Spacebar as fast as you can, just like in the previous task. When the dot will stop popping up, you will have to relax and keep listening to the audio track. Press Spacebar to continue."

Thereafter:

"Almost done! Now you will have to repeat the same task you did first: dot and sound will appear randomly. Respond as fast as you can by pressing Spacebar. Press Spacebar to continue."
